# The changing epidemiology of dog bite injuries in the United States, 2005–2018

**DOI:** 10.1186/s40621-020-00281-y

**Published:** 2020-11-01

**Authors:** Peter S. Tuckel, William Milczarski

**Affiliations:** 1 Department of Sociology, Hunter College, 695 Park Avenue, New York, NY 10065 USA; 2 Department of Urban Policy and Planning, Hunter College, 695 Park Avenue, New York, NY 10065 USA

**Keywords:** Dog bites, Dog-bite injuries, Dog owners, Epidemiology

## Abstract

**Background:**

In 2018, the most recent year for which data are available, dog bites ranked as the 13th leading cause of nonfatal emergency department visits in the United States. As dog ownership spirals upwards in the United States, it is important to continue to monitor the epidemiology of dog bite injuries. This study provides contemporary data on the incidence of dog bites injuries in the United States and in New York and profiles individuals who have been treated for dog bites in emergency departments. The study also examines the demographic correlates of the rate of injuries at the neighborhood level in New York City and maps the rate in each neighborhood.

**Methods:**

At the national level, the study examines longitudinal data on dog bite injuries from 2005 to 2018 gathered by the Centers for Disease Control and Prevention. For New York, the study analyzes data for 2005–2018 collected by the New York State Department of Health. A negative binomial regression analysis was performed on the state data to measure the simultaneous effects of demographic variables on the incidence of dog-related injuries. A thematically shaded map of the rate of dog bite injuries in New York City’s neighborhoods was created to identify neighborhoods with higher-than-average concentration of injuries.

**Results:**

In both the United States and New York the rate of dog-bite injuries increased from 2005 to 2011 and then underwent a significant decline. Injuries due to dog bites, however, still remain a sizable public health problem. Injuries are more prevalent among school-age children, inhabitants of less-densely populated areas, and residents of poorer neighborhoods. In New York City, poorer neighborhoods are also associated with fewer dogs being spayed or neutered.

**Conclusions:**

To reduce the rate of dog bite injuries, prevention programs – particularly those which center on teaching the dangers of canine interactions with humans – should be targeted at children. Dog bite injuries tend to be clustered in identifiable neighborhoods. Dog bite prevention programs and stricter enforcement of dog laws can target these neighborhoods.

## Background

While the appellation attached to dogs is “man’s best friend,” dog bite injuries are a common occurrence. Data covering the years 2001–2003 revealed that approximately 4.5 million individuals in the United States were bitten by dogs each year (Gilchrist et al. 2008). Of these, 19% necessitated medical attention. Between 2005 and 2013, there were an average of 337,103 visits to emergency departments (EDs) per year for dog bites (Loder 2019). In 2018, almost 27,000 individuals required reconstructive surgery owing to dog bites (American Society of Plastic Surgeons 2018).

The morbidity associated with dog bites is particularly pronounced among children. One study estimates that approximately one-half of all children aged 12 and younger have been bitten by a dog (Beck and Jones 1985). Of nine causes of injury resulting from activities children frequently engage in (e.g., baseball, playground accidents, etc.), dog bites rank second in terms of annual visits to an ED (U.S. Product Safety Commission 1996).

Most dog bite injuries in the US are inflicted by owned pet animals and not strays. Three of five bite victims were bitten by the family dog or one living in the neighborhood (Overall and Love 2001).

Significantly, the number of dogs in the United States has steadily increased over the last two decades. In 2000, approximately 68 million dogs were owned as pets in the United States. By 2017, the number of dogs had climbed to 89.7 million (APPA 2017). The total population of the United States in 2017 numbered 325 million. In terms of household penetration, 63.4 million households (49.3%) now own a dog (Insurance Information Institute 2019).

As the number of dogs has spiraled upwards, both the demographics of dog owners and the characteristics of the dogs themselves have undergone a noticeable change. During the past decade, the rates of dog ownership have risen sharply among older adults, Hispanics, and residents of large metropolitan areas. Correspondingly, there has been a decrease of dog ownership among families with young children (Marketresearch.com 2019). The share of dogs which are smaller (weigh under 25 pounds) has increased as well in this time span and this trend is expected to continue (PetfoodIndustry.com 2015).

With the growth in the ownership of dogs in the United States and the shift in the characteristics of both owners and dogs, it is important to continue to monitor the epidemiology of dog injuries and implications for public health. This study has five objectives: (1) to provide contemporary data on the incidence of dog bites in the United States and in New York, (2) to furnish a detailed profile of individuals who have been treated for dog bites in New York to describe individuals who are most at risk, (3) to present the socio-demographic correlates of the rate of dog bite injuries at the neighborhood level in New York City which can help to identify the characteristics of neighborhoods with a higher incidence of dog bite injuries, (4) to map the incidence of dog bite injuries at the local level which can be used to target neighborhoods which have a disproportionately large number of dog bite injuries, and (5) to provide data on the changing composition of the dog-owning population to help explain the epidemiological findings.

## Methods

### Data

The analyses conducted in this study rest principally on data collected from ED visits in the United States and New York. The national-level data are derived from the Web-based Injury Statistics Query and Reporting System (WISQARS) (Centers for Disease Control and Prevention 2019). WISQARS is an online, interactive database which provides national estimates of both fatal and nonfatal injuries. The present study utilizes the nonfatal injury data which comes from the National Electronic Injury Surveillance System–All Injury Program (NEISS-AIP), sponsored by the U.S. Consumer Product Safety Commission and the CDC’s National Center on Injury Prevention and Control. The NEISS-AIP is based on a sample of 66 hospitals randomly selected from all hospitals in the United States which have a 24-h ED and a minimum of six beds. The sample is stratified by hospital size measured in terms of the number of ED visits each year. The nonfatal injury data provide estimates of injuries treated in EDs by cause of injury (e.g., dog bites), race/ethnicity, gender, and disposition of the patient after being released from the ED.

In addition to the WISQARS database, this study examines individual-level patient records from New York. These patient records include a large number of demographic, diagnostic, and treatment variables. The patient records also include more detailed information concerning the racial and ethnic characteristics of patients than is contained in the national data sets. Importantly, the New York patient records include geographic identifiers such as the county or the 5-digit zip code in which the patient resides.

The data for New York come from the Statewide Planning and Research Cooperative System (SPARCS), which is under the auspices of the New York State Department of Health (2019). SPARCS assembles data on outpatient, inpatient, and ambulatory surgery patients treated in all hospitals in New York State.

This study also draws upon data gathered by New York City’s Department of Health and Mental Hygiene (DOHMH) (2019). The database consists of dog bites which are reported via online, fax, or phone to the city’s DOHMH Animal Bite Unit. Each record in the database provides information on: (1) the date of the bite, (2) the breed, age, and gender or the dog, (3) whether the dog was spayed or neutered, and (4) the zip code and borough of the person who was bitten. Altogether, there were 10,280 records spanning the years from 2015 to 2017. This tally clearly underrepresents the incidence of dog bite injuries in the city. The comparable number of dog bite injuries which were treated in EDs in the city during the period 2015 to 2017 totaled 44,947. Most likely, this disparity was because individuals who were bitten both had to know to contact and take the initiative to contact DOHMH. It should be noted, too, that the breed of the dog was missing on 15.4% of the cases and the zip code of the person who was bitten was missing on 26% of the cases.

### Variables

Injury Code. For both the national and state data sets, identification of patients who were treated for a dog bite was based on two separate injury codes. The International Classification of Diseases, Ninth Revision (ICD-9) External Cause of Injury code (E-code) E906.0 – Dog Bite – was utilized for the years prior to 2015. Both the ICD-9 E-code E906.0 and the ICD-10CM E-code W54.0XXA – Bitten by dog (initial encounter) – were utilized for the year 2015. Just the ICD-10CM E-code W54.0XXA was used for the years 2016–2018.

Sociodemographic Characteristics. Both the WISQARS and SPARCS data sets furnished information about the age and gender of patients. The SPARCS data sets also included two separate variables about the race and ethnicity of patients. A typology was created from these two variables with the following five values: “white, non-Hispanic,” “black, non-Hispanic,” “Asian, non-Hispanic,” “other, non-Hispanic,” and “Hispanic.” Importantly, the SPARCS database included the patient’s county of residence and his/her 5-digit zip code.

### Statistical analyses

To measure the combined effects of year, background characteristics (i.e., gender, age, race/ethnicity), and geographic location on the incidence of dog bites, we conducted a negative binomial regression analysis using the patient records from New York. A negative binomial regression analysis was performed instead of a Poisson regression due to overdispersion of the data.

The population-based counts of both the number of outpatients and inpatients who were bitten by a dog served as the dependent variable in this analysis. The predictor variables comprised the year, geographic location, and the demographic characteristics of the patients. Year was measured as an interval-level variable ranging in values from 1 (corresponding to the year 2005) to 14 (corresponding to the year 2018). To capture possible curvilinear effects of year on the incidence of dog bites, a multiplicative term created by squaring the year variable was also incorporated into the analysis. Geographic location was a dichotomous variable with a value of 1 indicating New York City and a value of 0 indicating New York State omitting New York City. Gender was also a dichotomous variable with a value of 1 indicating male and a value of 0 indicating female. The age variable consisted of 7 categories: under 5, 5 to 9, 10 to 14, 15 to 19, 20 to 44, 45 to 64, and 65 and older. The racial-ethnic background of patients was made up of 5 groups as mentioned above: non-Hispanic white, non-Hispanic black, non-Hispanic Asian, non-Hispanic other, and Hispanic.

Since it can be assumed that the risk of being bitten by a dog varies by population sizes, an offset variable was introduced into the analysis. The offset variable was created in two steps. First, population counts were tallied for each combination of year, geographic location, gender, age group, and racial-ethnic category. So, for example, one count might comprise non-Hispanic Asian females between the ages of 10 to 14 living in New York City in 2014. Altogether, this step yielded 1960 different counts. Next, natural log transformations were carried out on each of these counts.

To measure the demographic correlates of the rate of dog bite injuries at the county level in New York State (*N* = 62), a three-step process was undertaken. First, the number of both outpatients and inpatients were combined for each county for the year 2018 (the most recent year for which data are available). Second, these figures were divided by the population of each county to obtain an injury rate. Finally, the rates were correlated with an array of socio-demographic variables at the county derived from the American Community Survey 2014–2018 (5-Year Estimates) (U.S. Census Bureau 2018). These variables consisted of the following: (1) population density per square mile, (2) the racial-ethnic composition of the county, (3) median family income, (4) per capita income, (31) percent of families with income below the poverty level, (6) percent of the population 25 and over with a B.A. degree or more, (7) percent of the population with no health insurance, and (8) percent of the insured population with public health insurance.

A similar procedure was conducted to examine the socio-demographic correlates associated with dog bite injuries at the neighborhood level in New York City. For this analysis, the number of outpatients and inpatients were combined for each 5 digit zip code in New York City (*N* = 179). Next these figures were aggregated up to the United Health Fund (UHF) level (*N* = 42) and divided by the population of each UHF district to obtain an injury rate. These rates were then correlated with the same set of socio-demographic variables described above calculated for each UHF district.

### Spatial analysis

To determine the geographic distribution of patients injured by dog bites at the neighborhood level in New York City, a thematically shaded map of the injury rate by the United Health Fund (UHF) district in which the patient resided was created. A Global Moran’s I was computed to assess whether the spatial distribution of the residences of the patients was geographically clustered or dispersed.

## Results

### National data and trends

The rates of dog bite-related injuries by age and sex for the period 2005–2018 are presented in Table 1. Consistent with previous research findings, the data show that age is strongly related to the rate of dog bite injuries (Gilchrist et al. 2008; Weiss et al. 1998; Hoff et al. 2005; Quirk 2012; Holzer et al. 2019). The modal category is the age group 5 to 9, followed by the age groups 0 to 4 and 10 to 14. From the age group 15 to 19 and older, injuries taper off considerably. The data also reveal that gender is associated with dog bite injuries. Through the age of 14, the rate of males exceeds that of females by a wide margin. For the older age groups, the disparity between the gender groups narrows.

**Table 1:** Estimated Rates of Dog Bite Injuries Treated in an Emergency Department by Gender and Age Group in the United States: 2005–2018

	Both Sexes	Males	Females
Age Group	Rate^a^ (95% CI)	Rate (95% CI)	Rate (95% CI)
0 to 4	183.9 (156.4–211.5) *n* = 515,159	200.7 (169.3–232.0) *n* = 287,227	166.3 (141.9–1908.) *n* = 227,784
5 to 9	202.9 (176.2–229.7) *n* = 573,130	228.4 (198.1–258.7) *n* = 329,453	176.4 (151.6–201.1) *n* = 243,655
10 to 14	155.4 (133.8–177.0) *n* = 451,812	184.4 (158.4–210.5) *n* = 274,028	125.1 (107.2–143.0) *n* = 177,784
15 to 19	111.4 (96.2–126.5) *n* = 335,729	115.4 (99.8–131.1) *n* = 178,420	107.1 (91.1–123.0) *n* = 157,309
20 to 44	108.9 (94.6–123.2) *n* = 1,607,452	110.8 (97.0–124.7) *n* = 823,362	106.9 (91.6–122.2) *n* = 784,090
45 to 64	85.4 (72.0–98.9) *n* = 968,890	80.9 (69.0–92.8)*n* = 447,302	89.8 (74.4–105.2) *n* = 521,567
65 and over	58.7 (48.6–68.8) *n* = 355,358	58.2 (47.3–69.1)*n* = 153,379	59.0 (49.2–68.9)*n* = 201,979

^a^Rates are calculated per 100,000 spopulation.

**Fig. 1:**
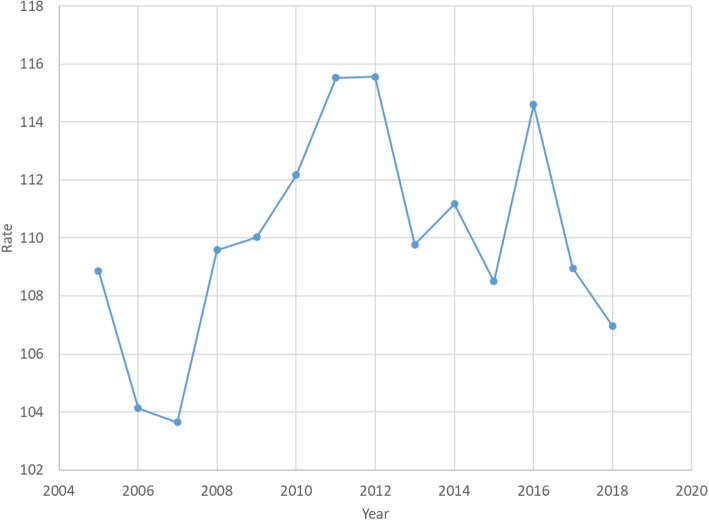
Annual Estimated Rate of ED Visits Due to Dog Bite Injuries in the United States (per 100,000 population)

Table 2 displays the annual estimated frequency and rates of dog bite injuries resulting in an ED visit in the United States from 2005 to 2018. Figure 1 graphs the estimated annual rates. The data show that the rates of injuries tended to increase until 2012 and then underwent an overall decline thereafter (*P* = .046 for the curvilinear relationship).

**Table 2:** Annual Estimated Frequency and Rates of ED Visits Due to Dog Bite Injuries in the United States, 2005–2018

Year	Frequency	Rate^a^
2005	321,694	108.9
2006	310,687	104.1
2007	312,231	103.7
2008	333,235	109.6
2009	337,526	110.0
2010	346,331	112.2
2011	359,972	115.5
2012	362,724	109.8
2013	346,925	109.8
2014	353,954	111.2
2015	347,952	108.5
2016	370,187	114.6
2017	354,154	108.9
2018	349,961	107.0

^a^Rates are calculated per 100,000 population.

**Fig. 2:**
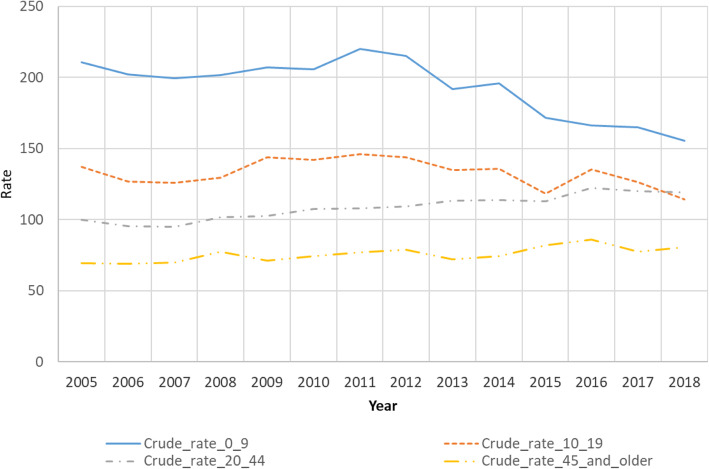
Annual Estimated Rate of ED Visits Due to Dog Bite Injuries in the United States by Age Group (per 100,000 population)

Noteworthy is that the relationship between the incidence of dog bite injuries varies by age group over time. Figure 2 exhibits the rates of dog bite injuries resulting in a visit to an ED by age group during the time period 2005 to 2018. Paralleling the overall trend for the country as a whole, the rates of the two youngest age groups (0 to 9, 10–19) initially increase up to 2012 and then undergo a steep decline. The opposite pattern prevails for the two older age groups (20–44, 45 plus). Here the rates of these two groups increase over the course of the 13 year span.

### New York State and New York City: individual-level effects

The results of the negative binomial regression analysis examining the simultaneous effects of time and key demographic variables on the incidence of dog bites treated in an ED are displayed in Table 3. The analysis is confined to two geographic locations – New York City and New York State excluding New York City. The predictor variables consist of year, year squared, place of residence, gender, age group, and the racial-ethnic background of patients.

**Table 3:** Negative Binomial Regression Estimates of Injuries Due to Dog Bites, New York State and New York City, 2005–2018

Variable	Exp(b)	95% CI
Time		
Year	1.086^**^	1.029–1.145
Year Squared	0.995^**^	0.991–0.998
Place		
New York City	0.714^***^	0.644–0.791
New York State minus New York City	(ref. category)	
Gender		
Female	0.895^*^	0.810–0.990
Male	(ref. category)	
Age category		
0 to 4	2.034^***^	1.681–2.462
5 to 9	2.821^***^	2.332–3.413
10 to 14	2.374^***^	1.961–2.873
15 to 19	1.991^***^	1.645–2.410
20 to 44	1.721^***^	1.424–2.080
45 to 64	1.501^***^	1.242–1.813
65 and older	(ref. category)	
Race/Ethnicity		
Non-Hispanic White	0.950	0.824–1.094
Non-Hispanic Black	1.060	0.919–1.221
Non-Hispanic Asian	0.460^***^	0.398–0.532
Hispanic	(ref. category)	

Significance level: ^*^ *p* < 0.05

^**^p < 0.01

^***^p < 0.001

The effects of year and the multiplicative term of year squared are both significant. A graphic display of these terms indicates that from 2005 to 2012 the frequency of dog bite injuries increased and then from 2013 to 2018 decreased, controlling for the other variables in the analysis. The same general pattern emerges if the injury rate of just individuals who were admitted as inpatients serves as the dependent variable. Both trends mirror the results observed at the national level.

Inspection of Table 3 indicates that residents of New York State outside of New York City are more likely to be treated in an ED for a dog bite than residents of the city. This finding reflects the greater prevalence of dog bite injuries in less densely populated areas. Coinciding with the findings from the national data discussed above, the table also shows that there is a significant gender gap in the incidence of dog-related injuries. Males are 1.12 times more likely to visit an ED due to a dog bite than females.

As expected, age is a major determinant of the risk of injury from a dog bite. Compared with patients who are 65 and older (the reference category), patients aged 5 to 9 are 2.7 times more likely to incur a dog bite injury and patients aged 10 to 14 are 2.3 more likely to sustain an injury. Individuals in the other age categories (0 to 4, 15 to 19, 20 to 44, and 45 to 64) are also significantly more likely to be injured by a dog bite than those in the reference category.

Finally, the data reveal that non-Hispanic Asians are considerably less likely to be treated in an ED for a dog bite than Hispanics (the reference category). The odds ratios for the other racial-ethnic groups are not statistically significant.

### Socio-demographic correlates of dog bite injury rate in New York State counties and New York City neighborhoods

Table 4 displays the relationship between key socio-demographic variables and the rate of injuries due to dog bites at both the county level in New York State and the neighborhood level in New York City. The data indicate that the rate of injuries due to dog bites is negatively associated with population density. This relationship between injury rate and population density is most pronounced at the county level. The data also show that at the county level, the injury rate is positively associated with the percent of the population which is non-Hispanic white, reflecting the relationship between urbanicity and racial-ethnic composition. Significantly, at both the county and UHF levels, there is a strong negative association between the injury rate and a number of economic variables. Injuries are more prevalent in counties or neighborhoods with lower median family income, per capita income, or proportion of the population which is not college-educated.

**Table 4:** Correlations Between Selected Demographic Characteristics and Dog Bite Injury Rate in New York State Counties and in New York City United Health Fund Districts: 2018

Demographic Characteristic	New York State Counties Correlation Coefficient (*N* = 62)	New York City United Health Fund Districts Correlation Coefficient (*N* = 42)
Population density (per sq. mile)	−0.35^**^	− 0.25
Percent non-Hispanic white	0.51^***^	−0.26
Percent non-Hispanic black	−0.50^***^	0.00
Percent non-Hispanic Asian	−0.46^***^	−0.11
Percent Hispanic	−0.42^**^	0.42^**^
Median family income^a^	−0.47^***^	−0.32^*^
Per capita income^a^	−0.43^**^	−0.34^*^
Percent of families below the poverty level	0.11	.48^**^
Percent of population 25 years of age and older who have a B.A. degree or more	−0.51^***^	−0.41^**^
Percent of population with no health insurance	0.07	0.26
Percent of insured population with public health insurance	0.35^**^	0.33^*^

^a^Median family income and per capita income were calculated by computing the median values of these two variables for all zip codes within each UHF district

Significance level: ^*^p < .05

^**^ *p* < 0.01

^***^ *p* < 0.001

Table 5 presents the results of an analysis performed on self-reported incidents of dog bites in New York City’s United Health Fund districts for the years 2015 to 2017.

**Table 5:** Correlations Between Selected Demographic Characteristics and (1) Percent of Dogs Who are Neutered/Spayed and (2) Percent of Dogs Which are Pit Bulls in New York City United Health Fund Districts: 2015–2017

Demographic Characteristic	Percent of Dogs who are Neutered/Spayed Correlation Coefficient (*N* = 62)	Percent of Dogs Which are Pit Bulls Correlation Coefficient (*N* = 42)
Population density (per sq. mile)	−0.01	− 0.19
Percent non-Hispanic white	0.82^***^	−0.72^***^
Percent non-Hispanic black	−0.57^***^	0.75^***^
Percent non-Hispanic Asian	0.35^*^	−0.58^***^
Percent Hispanic	−0.64^***^	0.45^**^
Median family income^a^	0.64^***^	−0.61^***^
Per capita income^a^	0.57^**^	−0.63^***^
Percent of families below the poverty level	−0.72^***^	.58^***^
Percent of population 25 years of age and older who have a B.A. degree or more	0.67^***^	−0.67^***^
Percent of population with no health insurance	−0.48^**^	0.25
Percent of insured population with public health insurance	- 0.73^**^	0.58^***^

^a^Median family income and per capita income were calculated by computing the median values of these two variables for all zip codes within each UHF district

Significance level: ^*^p < .05

^**^ *p* < 0.01

^***^ *p* < 0.001

The table shows the socio-demographic correlates of both the percent of dogs which were spayed/neutered and the percent of dogs which were pit bulls in the 42 UHF districts. Of the breeds identified in the data set (84.6%), pit bulls were the most numerous (33.6%), followed in order by Shih Tzu (5.3%), Chihuahua (5.2%), German Shepherd (4.1%), and Yorkshire Terrier (3.1%). This finding is consistent with previous research showing that pit bulls are responsible for more bites than any other dog breed (McReynolds 2019). Of the self-reported cases 29.1% were classified as spayed or neutered. The results reveal that poorer neighborhoods were associated with a higher proportion of dogs which had not been spayed/neutered and also a higher proportion of dogs which were pit bulls.

### Spatial distribution of dog bites in New York City’s neighborhoods

**Fig. 3:**
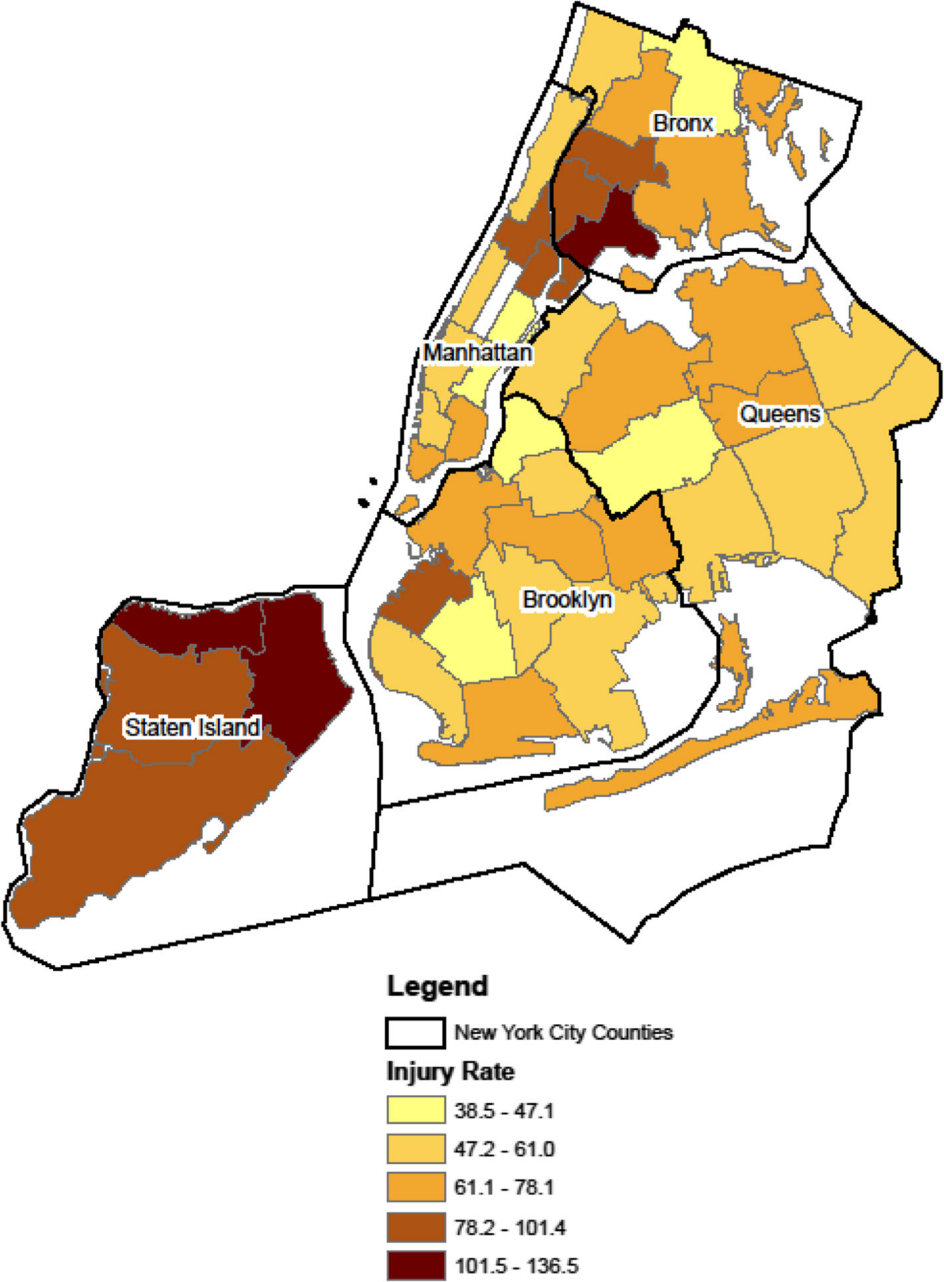
Rate of ED Visits Due to Dog Bite Injuries in New York City’s United Health Fund Districts (per 100,000 population)

Coinciding with expectations, the rates of dog-bite injuries are not uniformly distributed across the UHF districts. A choropleth map of the rates shows that the Hunts Point-Mott Haven neighborhood in the Bronx, East Harlem neighborhood in Manhattan, the Sunset Park neighborhood in Brooklyn, and the Port Richard and Stapleton-St. George neighborhoods in Staten Island have notably higher rates than other UHF districts (see Fig. 3). The Moran’s I Index yields a value of .356 (*p* < .001), indicating a pattern of spatial clustering .

### Profile of dog owners in the United States

Surveys of dog owners during the last decade reveal significant changes in their demographic characteristics (Table 6). The data in the table shows that the age distribution of dog owners has skewed upwards in the past decade. In 2008, 26.1% of dog owners fell into the age category of 55 to 74; by 2018, the number of owners in this age category rose to 31.5%. In this same 10-year span of time, dog owners were also more likely to be Hispanic, reside in larger metropolitan areas, and have higher levels of education. Another noteworthy change is the reduction in the number of younger children living in the household. The number of children in each of the age brackets under 6, 6 to 11, and 12 to 17 all dropped in the years from 2008 to 2018.

**Table 6:** Profile of Dog Owners in the United States: 2008–2018

Characteristic	Year		Percent Change
	2008	2018	
Age Group			
18–34	23.2%	24.1%	+ 0.9
35–54	45.1%	39.3%	−5.8
55–74	26.1%	31.5%	+ 5.4
75 and over	5.6%	5.1%	−0.5
Total %	100.0%	100.0%	
Race-Ethnicity			
White non-Hispanic	81.4%	75.5%	−5.9
Black non-Hispanic	4.9%	4.6%	−0.3
Asian non-Hispanic	2.1%	2.3%	+ 0.2
Hispanic	9.5%	15.1%	+ 5.6
Total %	100.0%	100.0%	
Location			
Top 25 Metro Areas	42.3%	45.8%	+ 3.5
Top 26–100 Metro Areas	40.9%	42.8%	+ 1.9
Not Top 100 Metro Areas	16.8%	11.4%	−5.4
Total %	100.0%	100.0%	
Education			
Non High School Graduate	12.5%	5.9%	−6.6
High School Graduate Only	28.7%	28.7%	0.0
Undergraduate College Degree Only	16.7%	20.7%	+ 4.0
Graduate Degree	7.3%	9.4%	+ 2.1
Total %	100.0%	100.0%	
Age of children in household			
Under 6	14.0	12.8	−1.2
6 to11	17.0	13.7	−3.3
12 to 17	15.8	12.8	−3.0
Total Number of Dog Owner Households (000 s)	42,010	46,962	+ 4952

## Discussion

This study has found that the rate of dog bite injuries has been declining in recent years. This decline is in evidence at both the national and state levels of analysis. The decline has been most visible among those under the age of 19 – particularly children under the age of 9.

One explanation for this downward trend might be that it is simply an artifact of the methodology employed in this study. Most of the findings contained in this study are based on dog bite injuries treated in emergency room departments. It may be the case, though, that in recent years individuals bitten by dogs have increasingly sought treatment in other venues such as private physicians’ offices or urgent care centers. While this may be a factor associated with the downward trend in dog bite injuries, another finding uncovered in this study -- a decline in the number of *inpatients* treated for dog bites in New York -- does not lend support to this explanation.

A second explanation for the recent decline in dog bite injuries centers on the change in the profile of dog owners and the characteristics of the dogs themselves. Survey data presented in this study indicates that there has been a decline in the presence of young children in dog-owning households over the past decade. Since young children are the most likely age group to be bitten by dogs and the overwhelming majority of injuries in the United States occur in the home, the reduction in the number of younger-aged children living at home would help to explain the drop off in dog-related injuries (Gilchrist et al. 2008; Weiss et al. 1998; Quirk 2012; Overall and Love 2001). The survey data further shows that dog owners are increasingly residing in larger metropolitan areas where dog sizes tend to be smaller. Also, the dogs are more likely to be confined indoors or in a yard, and kept on a leash (Marketresearch.com 2019). As research has demonstrated, small and medium size dogs pose less of a danger to humans and dogs on a leash lower the risk of unwanted contacts with humans.

The survey data also reveals that over the past decade owners have become older and better educated. This shift in age and education is consistent with the notion that the role of the dog has changed from being less of a guard dog and more of a pet or family member. A nationwide Harris Poll of adult Americans buttresses this notion. The poll found that among dog owners, 92% view their dogs as members of their family (Harris Poll 2011). Significant numbers report that they “allowed my pet to sleep in the bed with me” (70%), “bought my pet a holiday present” (60%), thought “it was a good idea to have dogs in long-term care facilities” to reduce stress (89%). The surge in the number of dog parks – designed to better meet the physical and emotional needs of dogs -- is another indicator of the changing role of the dog in American life. In the last decade there has been a 40% increase in dog parks, according to the Trust for Public Land (Lowrey 2020). Thus, it is likely that the changing role of the dog in U.S. also accounts for the lower incidence of dog bite injuries.

While there has been a diminution in the rate of injuries due to dog bites in recent years, dog bites still remain a leading cause of nonfatal injuries in the United States. For the year 2018 – the most recent year for which nationwide data are available -- there were a total of 344,201 nonfatal injuries treated in an ED due to dog bites.

As this study has noted, there is a distinctive socio-demographic profile of individuals who suffer an injury from a dog bite. Sizable age disparities exist, with younger individuals considerably more likely to be treated for a dog bite than older individuals, especially school-age children. Numerous reasons have been given to account for the greater susceptibility to dog bites on the part of young children. Children may lack the maturity to understand the “signaling behavior” of dogs, misinterpreting the cues dogs emit when in an agitated state. Children may also make sudden body movements or high-pitched sounds which can frighten dogs and precipitate an aggressive response (Overall and Love 2001). In addition, because of their “innate curiosity,” children may more readily approach strange dogs (Cohen-Manheim et al. 2018).

Aside from age, significant associations also exist between dog bite injuries and the place of residence and the economic background of patients. Dog bite injures are more prevalent among inhabitants of less densely populated areas and poorer neighborhoods. The negative relationship between the incidence of dog bites and the socioeconomic status of the neighborhood could be due to several factors. First, dogs might lack proper training or be taught to act aggressively to protect the household. Second, they may lack the necessary supervision by being chained outdoors for lengthy periods of time or being allowed to run loose. Third, as the data in this study has demonstrated, the owners may not be compliant with the licensing requirements such as spaying or neutering their dogs.

Though dog bites remain a sizable problem, it is one which is largely preventable. In this study we have found that dog bite injuries tend to be clustered in identifiable neighborhoods. Dog bite prevention programs as well as stricter enforcement of dog laws can target these neighborhoods to significantly reduce the incidence of injuries.

Some limitations attached to this study should be noted. One is that population counts of dogs and specific breeds within New York City neighborhoods are not available. Thus, we cannot determine the degree to which the varying prevalence of dog bite injuries in New York City’s neighborhoods is due to the differing number of dogs in these neighborhoods. Nor can we determine whether pit bulls rank as the most dangerous breed in New York City because of their attributes, or because of their numerical representation in the city. A further limitation pertains to the self-reported data on dog bites in the city. DOHMH states on their web page that “Data on breed, age, gender and spayed or neutered status have not been verified by DOHMH and is listed only as reported to DOHMH”. A third limitation pertains to the underlying reasons for the variability in the annual rate of dog bite injuries in the United States, particularly in the last several years. While we believe that this variability reflects a real diminution in the rate of dog bite injuries, we cannot dismiss the possibility that some of this variability might be due to data quality issues.

## Conclusions

This study has found that the rate of dog bite injuries in the United States has decreased in recent years. We attribute this decline mainly to a shift upwards in the age distribution of dog owners and to the changing role of the dog in American families from being less of a guard dog to being more of a companion. As dog ownership continues to spiral upwards (a trend which has been accelerated by the coronavirus and subsequent lockdowns), it will be important to monitor the frequency of dog-bite related injuries to see if this positive trend persists.

Though dog bite injuries have declined in recent years, the extent of these injuries still constitutes a major health problem. Young children especially are vulnerable to being bitten by a dog. Prevention programs – particularly those which center on teaching the dangers of canine interactions with humans – should be targeted at this age group. This study also has noted that residents of poorer neighborhoods in urban areas are more susceptible to being injured than residents of more affluent neighborhoods. Future research needs to be conducted to increase our understanding of why there is a negative association between a neighborhood’s socioeconomic status and injury rates from dog bites. Hopefully this greater understanding will lead to a reduction in the disparity of these rates.

## Data Availability

The WISQARS database can be accessed online (https://www.cdc.gov/injury/wisqars/nonfatal.html). Information about obtaining the SPARCS data can be obtained by contacting the New York State Department of Health (https://www.health.ny.gov/statistics/sparcs/). The DOHMH data can be retrieved online https://data.cityofnewyork.us/Health/DOHMH-Dog-Bite-Data/rsgh-akpg.

## Abbreviations

ED: Emergency Department
CDC: Centers for Disease Control and Prevention
DOHMH: Department of Health and Mental Hygiene
ICD-9: International Classification of Diseases, 9th Revision
ICD-10CM: International Classification of Diseases, 10th Revision
NEISS-AIP: National Electronic Injury Surveillance System-All Injury Program
SPARCS: Statewide Planning and Research Cooperative System
UHF: United Health Fund
WISQARS: Web-based Injury Statistics Query and Reporting System
